# Control of MnS Inclusions in High- and Low-Sulfur Steel by Tellurium Treatment

**DOI:** 10.3390/ma12071034

**Published:** 2019-03-28

**Authors:** Feng Wang, Hao Guo, Wei Liu, Shufeng Yang, Shuo Zhang, Jingshe Li

**Affiliations:** School of Metallurgical and Ecological Engineering, University of Science and Technology Beijing, Beijing 100083, China; b20150439@xs.ustb.edu.cn (F.W.); guohaoustb@sina.com (H.G.); youiithe@foxmail.com (W.L.); b20180496@xs.ustb.edu.cn (S.Z.); lijingshe@ustb.edu.cn (J.L.)

**Keywords:** MnS inclusion, Te treatment, sulfide inclusion, inclusion control, free-cutting steel

## Abstract

Manganese sulfide (MnS) inclusion in steel is related to the machinability of steel. Controlling the type and shape of MnS inclusion has been a problem for the steelmaking process for a long time. The effect of tellurium (Te) treatment on the control of MnS inclusion is studied with experiments and discussion of sequential solidification. The result shows that Te addition to the high- and low-sulfur steel dominantly changes the size and shape of MnS inclusions in steel. The aspect ratio of inclusions in cast steel changes with Te/S ranges from 0 to 1.9, which offers an effective way to control the MnS type and thus to improve the machinability and mechanical properties of steel. With an excessive Te addition, no further improvement could be observed by scanning electron microscopy-energy dispersive spectroscopy (SEM-EDS).

## 1. Introduction

Machinability is an important criterion in materials design for many applications. Free-cutting property is essential for some kinds of steel, which require lots of machining processes like gear steel and free-cutting steel [1,2,3]. So the inappropriate type of MnS will lead to the decline of mechanical property. According to Sims and Dahle [4], there are three types of MnS inclusions in cast steel. Type I, which is in a sphere shape, is said to be better for the mechanical property compared to the long, deformable type II and III MnS [5,6,7]. Therefore, the problem of controlling MnS type in steel is raised to balance the free-cutting and mechanical property, which is hard to realize because it is not easy to precisely control the small content of Mn and S in steel.

In order to control the sulfide morphology in the steelmaking process, methods of making the sulfide finer and dispersed or achieving the spheroidization of the sulfide are widely used. The former method is to use fine oxide inclusions as heterogeneous nucleation sites by adding titanium, magnesium, and zirconium [8,9,10], which is consistent with the concept of oxide metallurgy that fine and dispersed inclusions could improve mechanical properties of materials. The other method is modifying the morphology of the MnS inclusions by calcium or rare earth treatment [11,12,13,14]. Calcium treatment has been used to yield CaS inclusion, which has a high liquidus and will bond with MnS to form a compound that has higher liquidus than pure MnS [15]. The compound of CaS and MnS will crystallize earlier than pure MnS during the solidification of steel, which will improve the mechanical property of steel [16]. However the control of CaS content in steel is also a problem because it requires a very strict range of oxygen content for Ca treatment. If there are MnS and CaS inclusions in steel, it is difficult to achieve the desired result [17]. The addition of rare earth elements can purify the grain boundaries and achieve the effect of controlling the sulfide morphology. However, its high cost and strong ability to combine with oxygen limit its large-scale application in material production.

Tellurium is an element similar to sulfide because they are in the same main element group. However, Te is less active than S and O, which makes the reaction of Te with Mn more controllable [18]. Te treatment is different because Te combines with Mn instead of replacing Mn as compared to Ca. So it will not react with oxygen or sulfur. Many studies have pointed out that Te treatment could effectively change the morphology of MnS inclusions [18,19,20]. In the research, the experimental study of Te treatment was presented, and the effect of different Te additions to steel related to S content on the morphology of sulfide will be explored. The change of sulfide morphology in steel was studied by scanning electron microscopy-energy dispersive spectroscopy (SEM-EDS), and the suitable Te ratio in different sulfur content steels was discussed in theory compared to the experimental results. The S ratio and the mechanism of the change of sulfide during the cooling process were analyzed.

## 2. Experimental

### 2.1. Steel Content and Other Experimental Material

High-sulfur Steel 1 and low-sulfur Steel 2 are chosen to be added with 99.99% Te powder. The steel composition used for experiment is shown in Table 1. The Mn contents are high to make sure there are enough MnS inclusions in the steel.

### 2.2. Experimental Procedure

**Figure 1:**
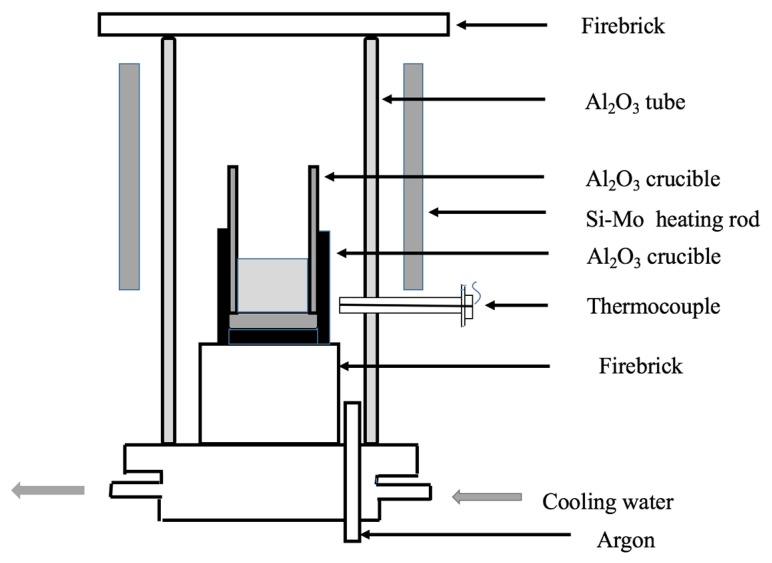
Schematic diagram of the heating furnace for the experiment.

The steel sample was first re-melted in a tube-type resistance furnace with argon atmosphere (0.3 m^3^/h) as shown in Figure 1. The furnace was kept for another 30 min to get homogenous content of liquid steel after heating up to 1600 °C. Then pure Te powder, which was wrapped up by iron foil, was added to the hot metal and then stirred with a molybdenum rod for about 15 s. The furnace remained at the temperature for 10 min and then the sample was taken out from the furnace with the condition of air cooling. The samples were cut from the ingot and made into small cube pieces (10 mm × 10 mm × 10 mm) and drillings for SEM-EDS (Phenom ProX, Phenom Scientific, Amsterdam, The Netherlands) and element detection of the ingot were made. The characteristics of the inclusions such as number density, size distribution, and aspect ratio were counted from the SEM images by the Image-Pro Plus software (6.0). The Te content was detected by the inductively coupled plasma mass spectrometry (ICP-MS) method. 

The experimental scheme is shown in Table 2. The scheme considers different combinations of Te and S content to make sure there are four types of Te/S ranging from 0 to 1 for high- and low-sulfur steel. High-sulfur Steel 1 and low-sulfur Steel 2 are defined as S1 series and S2 series steel samples.

## 3. Results

The contents of S and Te in each steel sample were tested and are shown in Table 3. It was a little different from the experimental scheme and showed a trend that with a high Te addition, the S content declined a little. Briefly the content varied a little from the planned scheme.

### 3.1. MnS Inclusion Morphology in Original Steel

**Figure 2:**
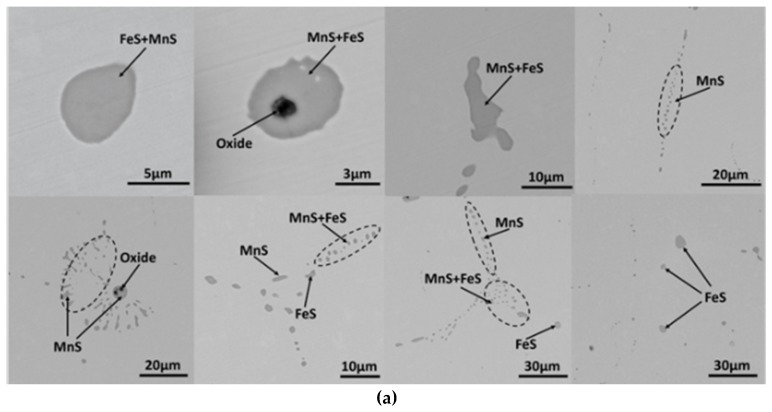

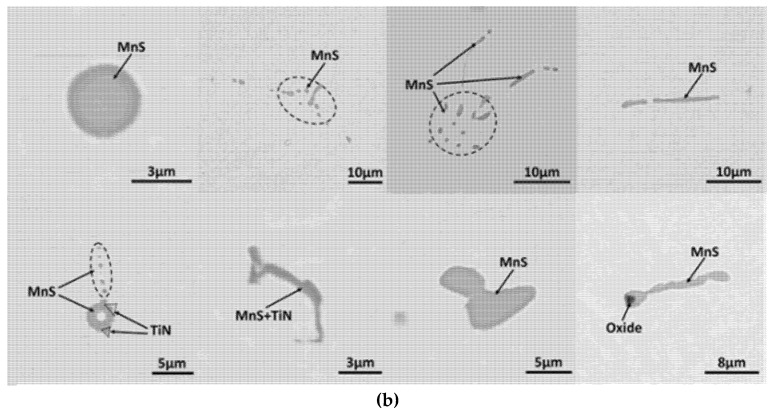
Morphology of typical MnS inclusion in original steel: (**a**) S1T0; (**b**) S2T0.

The morphology of MnS inclusion in S1 and S2 without Te addition is shown in Figure 2. It indicated that the original MnS inclusions in high and low sulfur are similar in shape, with both sphere and long strip shaped inclusions. The sphere inclusion accounted for about 52% of the total inclusion amount in S2T0 steel, while it accounted for almost 100% in S1T0 steel. However, the shape of MnS inclusions could not determine the type of MnS inclusions because the cutting process also affected the shape of MnS inclusion.

### 3.2. MnS Inclusion Morphology with Te Addition

**Figure 3:**
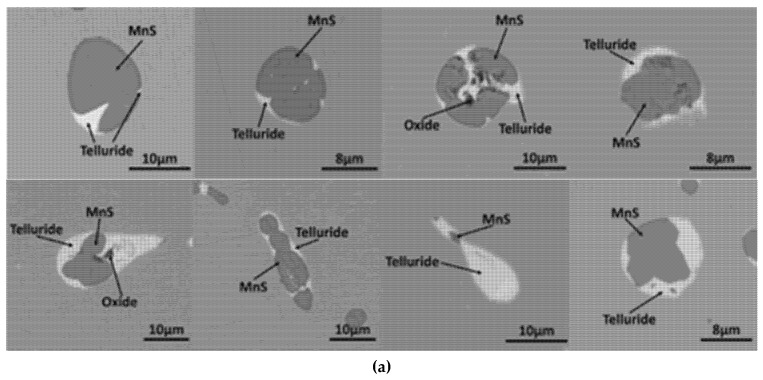

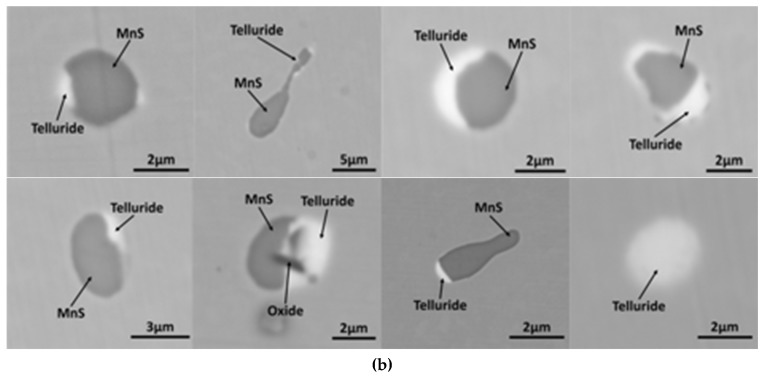
Morphology of typical MnS inclusion in steel with Te addition: (**a**) S1—high-sulfur steel; (**b**) S2—low-sulfur steel.

**Figure 4:**
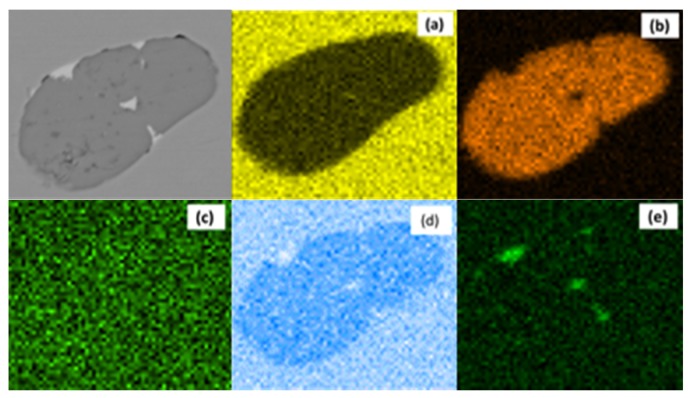
EDS map scanning result of typical MnS inclusion by Te treatment: (**a**) Fe; (**b**) S; (**c**) O; (**d**) Mn; (**e**) Te.

The typical morphology of MnS inclusion with Te addition in S1 and S2 steel is shown in Figure 3. Compared to the aforementioned results in Figure 2, inclusions with white covering start to show up in Figure 3; these were verified to be a compound with Te by energy dispersive spectrometer (EDS) scanning (Figure 4). The cross section of the inclusion from SEM observation shows that most MnS inclusions were wrapped by MnTe at the edge. There were few standalone MnTe inclusions and most of them were combined with MnS into duplex inclusions. Minor content of Te element was found in some MnS inclusions, and this result means that the MnTe was not only generating by attaching to the existing MnS, but also had the possibility of precipitating at the same time, which appears to be intertwining and co-existing in the SEM image. The MnS inclusion in high-S steel had a larger size than that in low-S steel even with Te treatment, which is consistent with the results in Figure 2. However, the shape of the inclusion shows some differences from Figure 2, namely, that after Te treatment, long strip MnS tended to be more rounded instead of having a sharp edge. The chain-like inclusion disappears in Figure 3. As for large MnS inclusions, MnTe grew and formed from the inside of them. Meanwhile for smaller ones, there was little MnTe growth from the inside and most of them formed a shell layer on the MnS inclusion.

**Figure 5:**
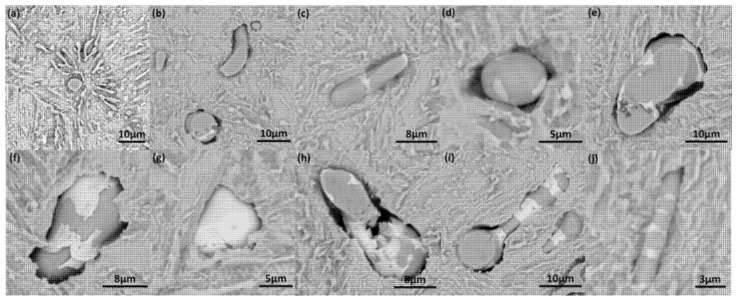
SEM image of MnS morphology of eroded S1 steel. (**a**) Before Te addition; (**b**–**j**) After Te addition.

The cross section cannot give a full view of inclusions. A steel sample eroded by 4% Nital observed by SEM shows more details in a three dimensional (3D) way (Figure 5). In Figure 5, the sphere inclusion appears to be long strip shaped in the 3D view. Therefore, the cross-section shape cannot totally represent the real shape of the inclusion. The white MnTe formed a shell layer on the MnS rod, and some even grew into the rod, which may lead to a break in the body of the MnS. The breaking of long rod-like MnS is good for the mechanical property of steel because of the small aspect ratio and dispersive spatial distribution of the inclusion.

**Figure 6:**
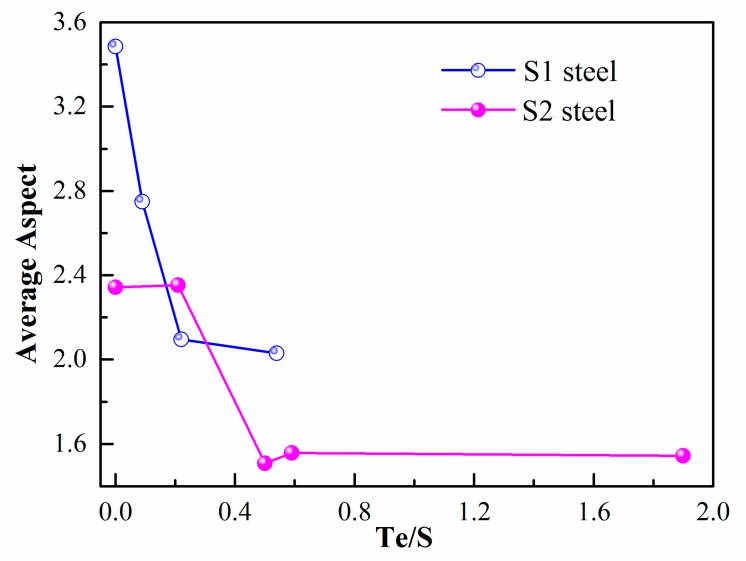
The average aspect ratio of inclusion changes with different Te/S in S1 and S2 steel.

Even though the 2D cross section of inclusion cannot indicate the whole morphology of MnS, Te treatment did have a significant influence on the inclusion characteristics. Figure 6 is the average aspect ratio of inclusions in S1 and S2 steel. After Te addition, the aspect ratio had a sharp decline, which proves the effect of Te treatment. The difference was that low-sulfur steel S2 had a decline of aspect ratio with a little amount of Te, while the S1 sample needed more Te to obtain the smallest aspect. Both steel S1 and S2 had the same reduction trend of the inclusion aspect ratio. Both of the steels showed that excessive Te addition would not improve the aspect but do no harm to it too. The best Te/S for Te treatment was about 0.5 for both low- and high-sulfur steel in this study.

## 4. Discussion

### Mechanism of Te Treatment

**Figure 7:**
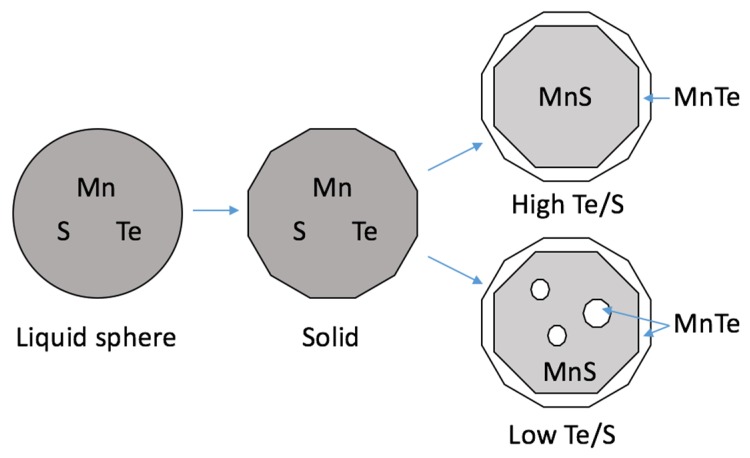
Schematic diagram of the generating process of MnS inclusions with Te addition.

It is obvious that Te addition can change the morphology of sulfide inclusions according to the results above. Some studies have presented the mechanism of this phenomenon [15,17,18,19]. It is said that type II sulfide precipitates as a second phase during the solidification of steel and distributes on the boundary of the grain. With Te addition, the precipitation temperature increases due to the chemical reaction of MnS with MnTe, which makes the cosolvent crystalize earlier than pure MnS. Small inclusions may form in the ferrite grain and have natural sphere shape for liquid inclusion instead of long strip shape because of the grain boundary. The liquidus of MnTe is lower than that of MnS, and during the solidification [21], it will precipitate from the cosolvent of MnS-MnTe and diffuse to the edge of the liquid inclusion and finally solidify as a shell wrapping the MnS core. With the condition of fast cooling, the Te cannot diffuse sufficiently, and this will lead to MnTe crystallization inside MnS, which looks like they intertwine with each other. This is good for distributing manganese inclusions and improves free-cutting and mechanical properties of steel at the same time. If there are some oxides with high liquidus to precipitate first, the crystallization of manganese inclusion usually takes the oxide as a nucleus. Since the MnTe always takes part in the shell of the inclusion, the interfacial property of the composite inclusion should be similar to pure MnTe, which is supposed to distribute more dispersedly than MnS. The schematic diagram of this reaction process is shown in Figure 7.

## 5. Conclusions

High- and low-sulfur steels were tested by Te-treatment experiments to indicate the effect of Te addition on the shape and size control of MnS inclusion to reach a balance between the machinability and mechanical property of steel. The characteristics of sulfide inclusions were detected by SEM-EDS and more detailed information of them was detected by other means such as transmission electron microscopy (TEM) and X-ray diffraction (XRD).

(1) Through the experiment, both in high- and low-sulfur steel, the aspect ratio and size changed dominantly with the addition of Te. The best Te/S to reach the lowest average aspect of inclusion was about 0.5 for both high- and low-sulfur steel in this experimental setup.
(2) The mechanism is discussed from a sequential solidification point of view. The MnS and MnTe were supposed to react as a second liquid phase and then, during its cooling, MnTe diffused to the shell while MnS remained in the core to form the core–shell inclusion.

## Figures and Tables

**Table 1:** Steel content used for the experiment.

Steel	Content	C	Si	Mn	P	S
1	wt.%	0.10–0.18	<0.15	0.8–1.2	0.05–0.1	0.23–0.33
2	wt.%	0.17–0.23	0.17–0.37	0.80–1.10	≤0.035	≤0.035

**Table 2:** Experimental scheme.

Heats	S, wt.%	Te, wt.%	Te/S	Heats	S, wt.%	Te, wt.%	Te/S
S1T0	0.3	0	0	S2T0	0.02	0	0
S1T1	0.3	0.015	0.05	S2T1	0.02	0.004	0.2
S1T2	0.3	0.06	0.2	S2T2	0.02	0.006	0.3
S1T3	0.3	0.15	0.5	S2T3	0.02	0.01	0.5
				S2T4	0.02	0.02	1

**Table 3:** S and Te content in steel samples.

Heats	S, wt.%	Te, wt.%	Te/S	Heats	S, wt.%	Te, wt.%	Te/S
S1T0	0.28	0	0	S2T0	0.020	0	0
S1T1	0.28	0.024	0.09	S2T1	0.020	0.0042	0.21
S1T2	0.26	0.057	0.22	S2T2	0.014	0.007	0.5
S1T3	0.26	0.14	0.54	S2T3	0.019	0.01	0.53
				S2T4	0.01	0.019	1.9
